# Governance Innovations for forest ecosystem service provision – Insights from an EU-wide survey

**DOI:** 10.1016/j.envsci.2022.02.032

**Published:** 2022-06

**Authors:** Carsten Mann, Lasse Loft, Mónica Hernández-Morcillo, Eeva Primmer, Francesca Bussola, Enzo Falco, Davide Geneletti, Ewelina Dobrowolska, Carol M. Grossmann, Giorgia Bottaro, Christian Schleyer, Tatiana Kluvankova, Gino Garcia, Marko Lovrić, Mario Torralba, Tobias Plieninger, Georg Winkel

**Affiliations:** a Eberswalde University for Sustainable Development, Chair for Sustainable Forest Resource Economics, Schicklerstrasse 5, Eberswalde 16225, Germany; b Leibniz Centre for Agricultural Landscape Research (ZALF), Eberswalder Str. 84, Müncheberg 15374, Germany; c Finnish Environment Institute, Latokartanonkaari 11, Helsinki 00790, Finland; d Forest Service of the Autonomous Province of Trento, via Trener 3, Trento 38121, Italy; e Department of Civil, Environmental, and Mechanical Engineering, University of Trento, Via Mesiano 77, Trento 38123, Italy; f Eberswalde University for Sustainable Development, Faculty of Forest and Environment, Schicklerstrasse 5, Eberswalde 16225, Germany; g Forest Research Institute Baden-Wuerttemberg (FVA), Department of Societal Change, Wonnhaldestr. 4, Freiburg 79100, Germany; h Land Environment Agriculture and Forestry Department (TeSAF), University of Padova, Viale dell’Università 16, Legnaro, Padova 35020, Italy; i Institute of Geography, University of Innsbruck, Innrain 52f, Innsbruck 6020, Austria; j Faculty of Organic Agricultural Sciences, University of Kassel, Steinstraße 19, Witzenhausen 37213, Germany; k SlovakGlobe: Slovak University of Technology and Slovak Academy of Sciences, Vazovova5, Bratislava 81243, Slovakia; l Eberswalde University for Sustainable Development, Schicklerstrasse 5, Eberswalde 16225, Germany; m European Forest Institute, Yliopistokatu 6B, Joensuu 80100, Finland; n Faculty of Organic Agricultural Sciences, Universität Kassel, Germany; o Department of Agricultural Economics and Rural Development, Georg-August-Universität Göttingen, Platz der Göttinger Sieben 5, Göttingen 37073, Germany; p European Forest Institute, Governance Programme, Platz der Vereinten Nationen 7, Bonn 53113, Germany

**Keywords:** Forest ecosystem services, Forest governance, Governance innovation, Enabling factors, European forests, Forest ownership

## Abstract

This paper analyses the occurrence of governance innovations for forest ecosystem service (FES) provision in the forestry sector in Europe and the factors that influence innovation development. Based on a European-wide online survey, public and private forest owners and managers representing different property sizes indicate what type of governance innovation activities they engage in, and why. To investigate forestry innovations as systems, the analysis focuses on biophysical, social and technical factors influencing innovation development. The results of our exploratory quantitative analysis show that most innovation activities identified are largely oriented towards biomass production. Accordingly, most forest owners implement efficiency-driven optimisation strategies for forest management and technological improvement for provisioning service supply, to generate income. In contrast, the provision of regulating and cultural services is not yet a prominent part of forestry innovation activities.Reasons are rooted in a market-oriented economic rationale focusing on timber production, a lack of financial resources to compensate for other FES provisions or institutions to provide backup and security to forest owners and managers for engaging in innovation development. Given that the provision of a wide range of FES is a politically well-established objective for forest management in Europe, a strategy is needed that helps to align actors and sectors for supporting and co-financing related forest management approaches and business models. The current revision of the forest related policy framework on EU level under the EU Green deal poses a window of opportunity for better fostering novel governance approaches for more sustainable FES provision.

## Highlights

• Sustainable provision of FES require a system- and innovation-based perspective.
• Most innovation activities are characterised by optimisation strategies for biomass provision.
• Lacking resources and institutions prevent foresters to engage in innovation activities.
• New forms of communication, cooperation and financing are needed for providing non-wood FES.
• Current revisions of EU policy frameworks pose possibilities for novel forms of FES governance.

## 1 Introduction

European forests have multiple functions and provide a range of forest ecosystem services (FES) to society (García-Nieto et al., 2013, Orsi et al., 2020, Saarikoski et al., 2015). Yet, how governance and innovation could effectively support the sustainable provision of FES has received less attention in forest science and policy. One reason is the traditional focus of professional forest management systems on increasing the efficiency of timber and biomass production (Nichiforel et al., 2020, Winkel and Sotirov, 2016). Indeed, biomass production focused management practices prevail in many cases, connected to economic incentives but also professional cultures of forest managers (Sotirov and Winkel, 2016), resulting in rather homogenous forest structures, even when policy goals are directed towards multi-functionality (Aggestam et al., 2020, Puettmann et al., 2012, Sotirov and Storch, 2018, Sutherland and Huttunen, 2018).

Coinciding with a primary focus on timber production and wood-based value chains, socio-political demand for the wide range of non-timber FES has steadily grown in recent decades, in particular for habitat provision, carbon sequestration and scenic beauty (Primmer et al., 2020, Ranacher et al., 2017). This has resulted in shifting focus in forest management approaches and policy objectives towards sustained flows of forest goods and services, beneficiaries’ values and ecological functions (Bauhus et al., 2017a, Grassi et al., 2017, Kleemann et al., 2020). Respective concepts of sustainable forest management and multifunctional forest management have been institutionalised as a core forest policy paradigm and practice in many European countries (e.g., Messier et al., 2019; Sotirov et al., 2014), seeking to integrate timber production with regulating and cultural ecosystem services (e.g., Borrass et al., 2017). However, to date, forest management decisions in most regions of Europe are heavily based on financial returns from timber production (as marketable products) and wood prices rather than the delivery of additional non-timber ecosystem services (Coll et al., 2018, Quine et al., 2013). Against this background it remains unanswered as to how novel and innovative ways of ecosystem service provision can be promoted and what context factors constrain or enable such innovations and vice versa.

Due to the public good character of many FES (e.g., Dwyer et al., 2015; Farley and Costanza, 2010; Nichiforel et al., 2018), the institutional complexity (e.g., Primmer et al., 2020; Sotirov and Arts, 2018; Winkel and Sotirov, 2016) and variation in forest ownership and forest owner goals (e.g., Ficko et al., 2019; Lawrence et al., 2020), governing the range of FES requires innovative approaches (e.g., Mann et al., 2021; Weiss et al., 2010). In the past decades, various governance approaches emerged throughout Europe that support the provision of non-marketable FES or bundles thereof. These include new silvicultural practices to more close-to-nature management or improving species mix (e.g., Bauhus et al., 2017a; Krumm et al., 2020; Puettmann et al., 2012), the establishment of collaborative forest owner associations (e.g., Bowditch et al., 2020; Primmer, 2011), the setup of certification systems and the design of payment schemes for ecosystem services (Živojinović et al., 2015, Prokofieva and Wunder, 2014), among others. Often these governance approaches emerge as pilot studies or independent business endeavors at local level (Maier et al., 2021). Some of them proved to secure conservation and social functions of forests, and were able to provide alternative income streams for forest owners (e.g., Živojinović et al., 2015), while for many other governance approaches a systematic evaluation of their design, implementation, and outcomes are missing (e.g., Baylis et al., 2016; Börner et al., 2020; Maier et al., 2021).

To date, a systematic empirical analysis of novel governance approaches for the sustainable provision of FES has not yet been carried out. As a large number of factors influence the effectiveness and outcomes of forest governance, we develop an integrated multi-disciplinary perspective. It combines concepts and methods of social-ecological and socio-technical systems analysis (e.g., McGinnis and Ostrom, 2014; Ostrom, 2011) as well as of innovation systems analysis (e.g., Asheim et al., 2011; Geels, 2011), and apply it in our empirical analysis of forest owner’s views on their FES provision and governance innovations as well as the factors conditioning these. We structure our analysis along four research questions:

1. What type of governance innovations exist in European forests?
2. What is the relation between governance innovation types and FES they address?
3. What factors are enabling or hindering the development of governance innovations?
4. What is the influence of forest ownership type and forest size on the development of governance innovations?

In the following section, we present our theoretical foundation conceptualizing forestry systems as complex social-ecological-technical systems that foster or hinder governance innovation development and outcomes through context conditions. Section 3 describes the empirical analysis and the applied method building on a European-wide online survey that addressed forest owners and managers. Section 4 reports the findings regarding innovations and the factors influencing FES provision. In Section 5, we discuss the potential and implications for the upgrading and upscaling of FES governance innovations in Europe. We conclude with implications for forest management, detailing policy and business recommendations as well as some guidance on future research in Section 6.

## 2 Theoretical foundation

### 2.1 Forest ecosystem services (FES)

Since the 1990 s, the concept of ecosystem services has been mainstreamed into science and policy, highlighting the essential role that ecosystems play in supporting both life and economic systems (e.g., Costanza et al., 1997; Daily, 2000; IPBES, 2018; Rasmussen et al., 2018). Since then ecosystem services frameworks and classification systems have been developed (Díaz et al., 2019, Millennium Ecosystem Assessment (MA), 2005). The Common International Classification of Ecosystem Services (CICES) (Haines-Young and Potschin, 2013) is widely acknowledged in science and policy, and employed in the EU initiative on Mapping and Assessment of Ecosystems and their Services (MAES) (European Commission (EC), 2014; Maes et al., 2012). For this analysis, we base our forest ecosystem services (FES) categorization on the CICES system, and test its inclusiveness and the consistency of its categories in relation to different innovation types (Annex B).

### 2.2 Governance innovation types

In this paper, we make use of pertinent governance and innovation frameworks to elaborate on innovations in general, and on governance innovation in particular for FES provision, and the influences for innovation establishment and development, especially related to transitions towards more sustainable resource uses (Geels and Schot, 2007, Lovrić et al., 2019, Smith and Stirling, 2010, Van Lancker et al., 2016). Innovation is understood as the process of making changes to something established by introducing something new (Van Lancker et al., 2016). The changes made can be gradual and incremental or radical and disruptive. Recent innovation scholars describe innovation as an iterative social process that takes place within given cultural, scientific, technological, and political configurations. These processes are open-ended rather than linear developments (Rip, 2012). Innovation is thus not a straight-forward, linear process that can be programmed or would lead to precisely defined results (Kuhlmann et al., 2010). Instead, innovations can be understood as a vision that requires general learning among actors to find pathways to realise the vision (Voß et al., 2009).

The sustainable provision of the range of FES going beyond timber and biomass production requires novel approaches of actor constellations and coordination that we frame in the following as governance innovations. Governance is about processes of organising interaction between societal and political actors and their interdependencies in a defined system (Kooiman, 2003). Actors and organisations are embedded in governance structures and their behavior is guided by institutions (North, 1991). Institutions are constellations of formal and informal rules that determine objectives, set standards, influence motivations and behavior, initiate or reduce conflicts, and resolve disputes among actors (Eden and Hampson, 1997, Ostrom, 2009). Institutions execute these functions towards innovation by means of hierarchies (e.g. new policies), markets (new market or business models), networks (e.g. public-private partnerships), or mixes thereof (hybrids) (Williamson, 2004). In particular, hybrid modes of governance that combine market, hierarchy and/or network components prove to be capable for the sustainable management of natural resources and to overcome social dilemmas (Kluvankova et al., 2021; Ostrom et al., 2011). As governance innovations we consider new rules and organizational arrangements resulting in novel forms of forest management that allow for a sustainable provision of FES, to improve income sources or to provide alternative benefit streams. These innovations include the establishment of new markets and payment schemes to generate value from FES as well as novel forms of collaborations, including means of communication, contracts and the inclusion of new users that foster improved value chains or bundles of provisioning, regulating, and cultural FES (Mann et al., 2021).

In summary, governance innovation in the context of this study refers to novel processes, products or services initiated by forest owners and managers that seek to improve the sustainable provision of FES types or bundles thereof. These innovations build upon particular governance mechanisms in form of hierarchies, markets, networks or hybrid forms to coordinate FES provision. With this conceptualisation in mind, we empirically elaborate what FES specific innovations exist in the European forestry contexts, their relation to FES categories, governance types and focus in an exploratory manner.

### 2.3 Forestry system interactions and conditioning factors

On a conceptual level, links between the provision of ecosystem services and governance have often been defined as social-ecological systems (e.g., De Groot et al., 2010; De Groot et al., 2012; Loft et al., 2016). The provision of FES is largely determined by biophysical conditions, such as climate, geography, forest conditions, and the past and present management decisions of the landowner or manager. The demand for FES, on the other hand, is determined by a set of socio-economic and political factors such as societal interests and institutions, actor constellations, and power relations amongst different groups and their capabilities to express and lobby for their FES demand. In addition, recent research into social-ecological systems has further recognised technology as a key component of a complex system, and key factor for effecting system resilience (Anderies, 2014, Folke et al., 2016, Redman and Miller, 2015). In this view, scholars highlight that society, technology and the environment are seen as co-constituted and interrelated entities, where technology mediates human-nature interactions and shapes the practices and consequences these relationships bring in time and space (Ahlborg et al., 2019). A fundamental function of technology is to enable, shape, transform and condition the physical and communicative interactions with the environment and other humans to increase efficiency, comfort or control, acting on the interface between humans and environment (Ahlborg et al., 2019). Technology development in return produces ambivalent social-ecological outcomes, gives rise to or prevents systemic pressures and impacts on ecosystems. Furthermore, technology transforms the exercise of power and societal interactions, which may lead to change (Smith and Stirling, 2010). To assess the crucial role of infrastructure, technical artefacts, and knowledge for systems change processes, conceptual inspiration comes from Socio-Technical-Systems (STS) research (e.g., Bijker et al., 2012; Borrás and Edler, 2014; Smith and Stirling, 2010). Guiding this strand of research is a (quasi-)evolutionary understanding of technological change which regards technological innovation as an open-ended process, shaped in and shaping interactions between various actors and stabilizing gradually over time (Geels and Schot, 2007). These close interactions and interdependencies between societal and environmental systems and the intermediary and influential role of technology pledges for a conceptual understanding of forestry innovation systems that make use and combine social-ecological and socio-technical systems approaches (Ahlborg et al., 2019, Smith and Stirling, 2010).

In this vein, we understand forest management systems in which innovations for FES provision develop as social-ecological-technical systems (Sorge and Mann, 2018). They provide particular conditions that are shaped by biophysical, social (institutions and actors), and technical conditions (infrastructures, knowledge) that can enable or hinder innovation development. These forestry systems are complex, dynamic and multiscalar, nested in larger systems, and influenced by external factors, such as EU legislation or climate change. Taking on a system-based innovation understanding helps us to gain a more comprehensive picture on innovation establishment, in particular regarding the type of innovation, their relation to FES provision as well as regarding how innovation develops and what factors condition its emergence in a forestry context.

## 3 Material and methods

### 3.1 Survey design

To analyse the factors influencing FES supply and the factors influencing their pertinent governance innovations, we conducted a European-wide online survey administered to private and public forest owners and managers using Maptionnaire software.^1^ The survey was promoted by two H2020 Innovation Actions on novel policies and business models for the sustainable supply of forest ecosystem services (SINCERE and InnoForESt) (see Annex A for the full survey). This paper reports the responses concerning FES governance innovations overall, according to ownership type and forest size, independent of their geographical distribution and other demographic characteristics. A filter question selected respondents who stated to have implemented a FES-related governance innovation within the last two decades. It was followed by six closed-ended questions (Table 1).

**Table 1:** Survey questions and their variables.

	Question	Variable	Type / measurement
Q1	What type of forest ownership are you representing?	Land Tenure	Nominal / Multiple choice
Q2	Please state the size of the forest you own or are responsible for.	Forest size	Continuous / Whole number [ha]
Q3	Please describe [the following] ecosystem services in view of: a) those your forest area currently provides, and b) what societal demand for these services you perceive.	FES supply	Continuous scale / independent
Q4	In relation to your forests, has there been (such an) innovation for at least one ecosystem service in the last two decades?	Presence of Innovation	Binary
Q5	Which innovations have you developed? [choice of 10] Please also separately mark the most economically important one, and the most innovative one.	Economic and innovative relevance	Binary / dependent
Q6	To what extent do the following 15 factors support or constrain the innovations you have been developing?	Influencing factors enabling and hindering innovation	Continuous scale / Independent

### 3.2 Variables, data selection, and statistical analyses

#### 3.2.1 Governance innovation types in European forests

To analyse general trends of FES provision and specific governance innovations types that are developed by forest owners and managers across Europe, we only used datasets from respondents who answered ‘yes’ to question 4 (Q4) ‘In relation to your forests, has there been such an innovation for at least one ecosystem service in the last two decades?’. For an overview, we applied descriptive statistics including frequencies to derive information about the statistical distribution of innovation types, objectives, and influences.

For the investigation of implemented governance innovations, ten specific innovations were offered for selection to forest owners and managers (Q5: ‘Which innovations have you developed?’). These were supplemented with descriptive examples, for example, Q5_1 ‘New ecosystem service (e.g., a pollination strip or burial forest was newly established)’. Table 2 shows how specific innovations are linked to the conceptual orientation of the survey design referring to the FES categories they address, the governance innovation type, as well as type of innovation as described in Section 2.2.

**Table 2:** FES specific innovations and their relation to FES categories, governance innovation type, and focus.

Q5	Specific innovations offered for selection	Short name	Example provided in the Survey	Main FES categories addressed	Governance mechanism	Focus of innovation
Q5_1	New ecosystem service	New ES	e.g., a burial forest was newly established	Provisioning, Regulating, Cultural	Hierarchy, Market, Hybrid	Product, Service
Q5_2	New technology for biomass production	Technology biomass	e.g., usage of harvester instead of chainsaws or using satellite imagery for identifying logging sites	Provisioning	Market	Process
Q5_3	New technology for other ecosystem services	Technology other ES	e.g., a new technology for extracting resin	(mostly) Provisioning	Market	Process
Q5_4	New way to generate value from ecosystem services	Value from ES	e.g., organizing auctions for high-quality timber or water protection	Provisioning, Regulating	Market	Process, Service
Q5_5	Change of forest management to improve / sustain biomass production	FM for biomass	e.g., new thinning measures for increased wood increment or for increased resilience	Provisioning, Regulating	Market	Process
Q5_6	Change of forest management to provide other ecosystem services	FM other ES	e.g., new thinning measures for growth of mushrooms or support nature tourism	Provisioning, Regulating, Cultural	Market	Process
Q5_7	New communication or marketing strategy implemented	New communication	e.g., a website or a hired branding professional	Any	Market	Process
Q5_8	New users of ecosystem service(s)	New users	e.g., children or urban citizens	Any	Network, Hybrid	Process
Q5_9	New trans-sectoral contract created	New contract	e.g., a new agreement with conservation groups or eco-tourism enterprises	Regulating, Cultural	Network, Hybrid	Process
Q5_10	New transboundary cooperation created	New cooperation	e.g., a sustainable tourism project across country borders	Regulating, Cultural	Market, Network, Hybrid	Process, Product, Service

#### 3.2.2 Relation between FES and governance innovation types

The relationship between perceived supply of FES and governance innovations was analysed using answers to question 3 relating to ecosystem service provision and demand (see Table 1) with a scale ranging from ‘not supplied/ demanded by society’ to ‘very much supplied/ demanded by society’ (see Annex C “Conversion of continuous scale (1−100) to a 7-point Likert scale”). Based on the classes generated, values in the range 44–57 (value 4 on the Likert scale) were excluded from subsequent correlation analyses, to concentrate on the more meaningful values.

The addressed FES were analysed by calculating means for each FES supplied or societally demanded, and tested for normal distribution of individual variables (Kolmogorov-Smirnov test) with the use of histograms (see Supplementary Material Table S2 and S3). The distribution of variables relating to the 11 surveyed groups of FES was non-normal. Usually, more observations were found above the mean. Because a transformation of the continuous scale from the survey was made, a reliability analysis was performed to check whether the 7-point Likert scale is equivalently suitable to measure specific FES. This scale reliability was tested using Cronbach Alpha measurement, which in case of FES sub-categories indicated a scale consistency α = 0.812 (n = 11). It is assumed that a Cronbach Alpha value ≥ 0.7 indicates a reliable and acceptable scale (Taber, 2018). By means of a correlation analysis, we then explored the relationship between perceived supply and societal demand of FES. Based on very high correlations for most FES, we decided to consider only the perceived supply for testing their relationship to governance innovation types.

In order to reduce the dimensionality and complexity of supplied FES variables, and to check whether new factors would emerge from inter-correlated items that significantly differ from the CICES classification, we carried out an exploratory factor analysis (Principal Axis Factoring Method) with Varimax rotation. We thereby identified FES categories, i.e., provisioning, regulating, and cultural FES (see Table 6) as perceived by forest owners and managers and later compared them with the CICES categories. We probe the allocation of specific FES to the CICES categories provisioning and cultural FES, as a different allocation may explain differences in governance innovation for their provision. The procedure of exploratory factor analysis includes also prior inspection of the power of the relationships and factorability of the variables involved in the analysis (Beavers et al., 2013). The suitability of the questionnaire data for factor analysis was tested. A first test, the Bartlett’s Test of Sphericity checks whether there is or isn’t a certain redundancy between items analyzed that could be interpreted as a factor later on. It compares the observed correlation matrix of variables to the identity matrix, and checks if they are both the same. The sample adequacy was then checked with the KMO (Kaiser-Meyer-Olkin) Test that measures the degree of common variance among items selected for the factor analysis. Both tests revealed that the sample is adequate for the factor analysis (KMO = 0.799) and the Bartlett’s test was significant (Bartlett's test of sphericity p = 0.000) p < 0.05 which confirmed that the correlation matrix differs from the identity matrix so the factor analysis is proper to use.

In factor analysis, it is crucial to determine the number of factors that will best represent the whole data set. The goal is to select only those factors that are representative and theoretically adequate (Beavers et al., 2013, Fabrigar et al., 1999). We based our selection on Eigenvalue criteria (Eigenvalue > 1), scree plot, and the percent of variance explained by each factor. The final decision should take into account the interpretability and accuracy of the selected factors (Beavers et al., 2013). Therefore, initially the three, four, and five-factor solutions were investigated. Due to the highest total variance explained, clear factor loading values, and better comprehensibility the four-factor solution was chosen. The point-biserial correlation was run to determine the relationship between the resulting factors, respectively FES categories, and the governance innovation types being developed.

#### 3.2.3 Conditioning factors enabling or hindering governance innovations

In order to understand the reason why some governance innovations emerge more often than others, we were interested in the conditioning factors that influence, i.e. enable or hinder the emergence and development of innovations in the forestry sector. For analysis, responses to question 6 (Q6) ‘To what extent do the following factors support or constrain the innovations you have been developing?’ form the basis. Respondents could select the degree to which 15 predefined factors (Table 3) are supporting the respective innovation ranging from ‘very strongly not supporting to very strongly supporting’. Similar as for question 3 the 1–100 scale was converted into a 7-point Likert scale to allow for a better interpretation of the results (see Table C in the Appendix).

**Table 3:** Overview of potential influencing factors for governance innovation development offered in the survey, their system dimensions, and their codes used for the visualization of results.

Q6 Factor codes	Factors conditioning the emergence of governance innovation	System dimension
Q6_1	Regulatory framework (laws and rules)	Institutional (Social)
Q6_2	Policy makers and stakeholders	Actors (Social)
Q6_3	Private sector and business	Actors (Social)
Q6_4	Societal demand for the ecosystem service	Actors (Social)
Q6_5	High profitability/viability before the innovation happened	Markets (Social)
Q6_6	Low profitability/viability before the innovation happened	Markets (Social)
Q6_7	Profitability of the innovation	Markets (Social)
Q6_8	Abundance of ecosystem services	Biophysical (Ecological)
Q6_9	Scarcity of ecosystem services	Biophysical (Ecological)
Q6_10	Knowledge available	Technical
Q6_11	Public financial support (e.g., subsidies)	Markets (Social)
Q6_12	(Access to) private investment capital	Markets (Social)
Q6_13	Culture of your organization	Institutional (Social)
Q6_14	Individual leadership	Actors (Social)
Q6_15	Climate change	External

These variables were tested against the normal distribution with the use of the Kolmogorov Smirnov test. Histograms were produced, for the 1–7 (without neutral values) and for standardized values 0–1 (see Supplementary Material Table S5 and Table S6). None of the variables confirmed a normal distribution of the data. The peak of the observation distribution was always on the extreme side of the scale (close to 1 or close to 7). The reliability of answers re-coded to the 7-point Likert scale was crosschecked by conducting the Cronbach Alpha test. The test indicated that the new Likert scale assumed for 30 variables reached acceptable reliability (Cronbach’s Alpha = 0.819).

To identify factors that influence the development of governance innovations by forest owners most, the distribution of answers over all respondents and the mean values of perceived influence of these factors were analysed. Internal correlation between factors influencing the self-perceived “most economically important” and the “most innovative innovations” was tested. A correlation matrix was developed to test the governance innovation types against the given influencing factors. Therefore, we calculated confidence intervals based on random sampling with a replacement (bootstrapping) of the survey responses (for all variables of Q3 and Q6). They represent confidence intervals that are data-specific and thus more realistic than the ones usually obtained – i.e. pre-sampling confidence intervals where the distribution of responses is unknown and thus assumed to be normally distributed.

#### 3.2.4 Influence of forest ownership types and size

We explored how forest size and ownership type influenced the development of innovations in general (Q4), and the implementation of specific governance innovation types (Q5) in particular. Respondents could select one out of six predefined options for different types of private and public forest ownership (see Supplementary Material Tables S8 and Fig. S3 and S4). An indication of the size of the forests under their responsibility was requested in hectares (ha) offering continuous values. Respective frequencies were examined together with the data distribution and the results of a correlation analysis, considering all types of governance innovations implemented or not.

All analyses were run with SPSS 26 and R (RStudio). Graphs and tables were prepared with MS Excel. All graphs and tables produced are stored in the Supplementary Material.

#### 3.2.5 Survey distribution and sample description

The survey was distributed by umbrella organisations representing different types of forest owners and managers. These include the European State Forest Association (EUSTAFOR), the Confederation of European Forest Owners (CEPF), the European Landowners Association (ELO), the European Network of Forest Extension Organizations (FOREXT) and the European Federation of organizations representing forest municipalities (FECOF). These organisations represent all segments of the forestry sector on European and national levels. The pyramid and snowball sampling (Atkinson and Flint, 2004) allowed us to reach as many different types of forest owners and managers in different countries as possible. The survey targeted members of these umbrella organizations on national levels, who have again promoted the survey in their annual meetings, websites, newsletter and emailed survey distribution materials to their national member organizations who have further distributed the survey through their websites, newsletters and emailing lists in their respective native languages. To accommodate for such a mixed-mode mail and web distribution (Dillman et al., 2014), the survey was translated to 19 languages. The survey was first pre-tested within the participants of the SINCERE and InnoForESt projects and by representatives of all umbrella organizations that were to organize the survey’s distribution. Besides some changes in wording and the introduction of a more thorough explanation of different innovation types, the main changes resulting from the pretest of the survey was the removal of questions that were regarded as potentially sensitive to respondents, such as income, age and gender. The distribution started on 19.09.2019 and ended on 10.12.2019. In total, 1234 forest owners and managers participated in the survey. Among them, 467 participants (37%) stated that they had developed a FES related innovation (Q4). Of these 467 respondents, 101 respondents did not further detail the innovations developed (Q5). The final dataset of respondents who implemented a specific innovation comprised 366 cases which in sum developed a total of 1114 innovations and were the target of our analysis.

The distribution mode chosen does not address a defined number of individuals within a population and consequently does not allow to infer the return rate of the final sample but a description of the comprehensiveness of the targeted variables. Respondents from 17 European countries participated in varying numbers. Germany was the most represented country (33%), followed by The Netherlands (18%) and Finland (14%) according to the language chosen by the respondents. In the dataset, the six distinguished forest ownership types were also distributed unevenly. The majority of respondents identified with ‘Private ownership by individuals or families’ (76%) and ‘Public ownership by local government (municipality or equivalent)’ (11%), while ‘Public ownership by state at subnational, regional level’ (3%) and at national level (2%) was chosen least by respondents. Regarding forest size, small forest properties (< 20 ha) had a much higher representation (35%) than large forest properties (> 5000 ha; 8%), with half of the respondents owning or managing properties less than 60 ha (Torralba et al., 2020a).

## 4 Results

### 4.1 Governance innovation types in European forests

Governance innovations indicated by respondents were mostly developed for the improved provision of biomass (wood). Most prominently, ‘Change of forest management to improve/sustain biomass production’ and the use of ‘New technology for biomass production’ together represent 34.8% of total governance innovation types, while ‘Changes of forest management to provide other FES presented’ and ‘New technology for other ecosystem services’ represented only 15.1% of total governance innovation types (Table 4). The innovations directly related to biomass provision are considered the most economically important and innovative ones.

**Table 4:** Governance innovation types developed by forest owners.

Governance innovation type	The most economically important^a^	The most innovative	Total Innovations developed	% of Innovations developed
Change of forest management to improve/sustain biomass production	58	25	236	21.2
New technology for biomass production	67	37	151	13.6
Change of forest management to provide other ecosystem services	34	27	134	12.0
New way to generate value from ecosystem services	33	11	108	9.7
New users of ecosystem service(s)	20	15	108	9.7
New ecosystem service	28	32	107	9.6
New trans-sectoral contract created	22	21	99	8.9
New communication or marketing strategy implemented	19	18	86	7.7
New transboundary cooperation created	15	15	50	4.5
New technology for other ecosystem services (than biomass production)	14	13	35	3.1
TOTAL	310	214	1114	100.0

a Number of governance innovations stated

### 4.2 Relation between FES provision, supply and governance innovation types

Comparing the correlation between supplied and demand FES, the majority of respondents indicated that their forests mainly supplied wood-based provisioning services. This was the FES perceived by forest owners and managers as being most demanded by society, based on the question “Please describe what ecosystem services in your view your forest area currently provides, and what societal demand for these services do you perceive” (Table 5). In general, the inter-item correlation analysis confirmed that the perceived demand and supply for each FES variable were highly correlated (Table S1, supplementary material). Comparing the mean value given to each FES by respondents, the supply of seven FES was perceived greater than the demand, in particular for the three regulating FES ‘Habitat for plants and animals’, ‘Air quality regulation’, and ‘Climate change mitigation’. In contrast, ’Biomass for material and energy’ was perceived as having a balanced supply and demand while ’Education’ and ’Healthcare, sports and outdoor recreation’ were perceived as in higher demanded by society than currently supplied. All correlations are significant at the 0.01 level (2-tailed), p-value < 0.01.

**Table 5:** Perceived FES supply and demand ranked on the basis of the correlation values.

	Mean			Correlation
FES sub-categories	supplied	relation	demanded	Supplied vs. demanded
Wild forest products	43.56	<	51.41	0.642
Biomass (wood) for material	66.92	>	64.29	0.641
Biomass (wood) for energy	59.50	~	60.61	0.606
Cultural, emotional and spiritual values	64.55	>	57.93	0.605
Education	48.82	<	54.09	0.590
Game (hunting)	61.39	>	57.22	0.562
Healthcare, sports and outdoor recreation	62.04	<	66.72	0.551
Watershed protection	63.07	>	60.96	0.487
Air quality regulation	71.37	>	65.29	0.418
Climate change mitigation	77.99	>	70.73	0.320
Habitat for plants and animals	80.53	>	69.35	0.298
Valid N (listwise)	366			

The conceptual allocation of the FES sub-categories using factor analysis is presented in Table 6. The resulting factors largely correspond with the CICES FES categories but with two exceptions: regulating services (explaining 18% of the total variance), provisioning services (12%), cultural services (12%), and ’Wild forest products’ as an extra provisioning service category (6%) while ‘Cultural, emotional and spiritual values’ were not included in any of these factors or categories. Altogether, they explained 48% of the variance. Table 6 displays all factor loadings, where significant factor loadings that contributed the most to specific FES categories are in bold.

**Table 6:** Four-factors of forest ecosystem services, based on ‘perceived supply’ data.

FES sub-category	Regulating FES	Provisioning FES I (biomass and game)	Cultural FES	Provisioning FES II (other wild forest products)
Climate change mitigation	**0.779**	0.220	0.093	0.033
Air quality regulation	**0.740**	0.041	0.144	0.142
Habitat for plants and animals	**0.541**	0.152	0.318	0.003
Watershed protection	**0.490**	0.299	0.270	0.282
Biomass (wood) for material use	0.194	**0.764**	0.072	-0.041
Game (hunting)	0.062	**0.576**	0.131	0.230
Biomass (wood) for energy use	0.117	**0.511**	0.124	0.161
Education	0.121	0.146	**0.761**	0.096
Healthcare, sports and outdoor recreation	0.290	0.153	**0.627**	0.103
Wild forest products	0.101	0.199	0.114	**0.634**
Cultural, emotional and spiritual values	0.382	0.069	0.381	0.304
Eigenvalue	3.965	1.414	1.105	0.927
Explained variance (%)	18.160	12.981	12.471	6.312

Significant factor loadings are in bold (n = 366, p = 0.000)

The relationship between the governance innovation types and FES categories was then tested with a correlation matrix, using the FES factor scores derived from the Factor Analysis (Table 7). Significant correlations were found between the governance innovation type ‘New ecosystem services’ (Q5_1) and all four FES categories in a range from rpb = 0.137 to.208, p = 0.000. The correlations confirmed that the developments of a ‘New technology for biomass production’ were linked to ‘Provisioning FES I (biomass and game)’ (rpb = 0.224, p = 0.000).

**Table 7:** Significant correlations between governance innovation types and FES categories.

* Correlation is significant at the 0.05 level (2-tailed); ** Correlation is significant at the 0.01 level (2-tailed)

Correlation based on the results from Pearson’s correlation matrix (n = 366): measured as a point-biserial correlation matrix between the factors obtained in Factor Analysis (Table 6) and the governance innovation types (Table 4).

A significant negative correlation was found only between the ‘Change of forest management to provide other ecosystem services’ and ‘Provisioning FES I (biomass and game)’ (rpb = −0.119, p = 0.023). The governance innovations ‘New users of ecosystem service(s)’, ‘New trans-sectoral contract created’, and ‘New transboundary cooperation created’ correlated significantly only with ‘Cultural FES’ (rpb = 0.168 to.188). No significant correlation was found for ‘New way to generate value from ecosystem services’ or ‘Change of forest management to improve/sustain biomass production’ with any of the FES categories, therefore omitted in Table 7. The category ‘Cultural FES’ was the one most significantly correlated with governance innovations especially with ‘New ES‘ and ‘New ways of communication and cooperation’. ‘Wild forest products’ was identified as a stand-alone category, rather than belonging to provisioning FES as defined in CICES. It correlated significantly with genuinely ‘New ecosystem service’; ‘New communication or marketing strategy implemented’ and especially with the need for a ‘New technology for other ecosystem services (than biomass production)’ indicating their individuality compared to other ES. The complete correlation matrix with all variables and exact significance values can be found in the Supplementary Material, Table S9.

### 4.3 Factors conditioning the development of governance innovations

Several conditioning factors analysed appeared to influence innovation development. Table 8 presents the significant correlations between enabling/hindering factors for the self-perceived most economically important governance innovation types. To analyse whether factors were perceived as enabling or hindering innovation development, the mean value for each factor was calculated (see Supplementary Material Table S4). They range from 3.13 for ‘Low profitability/viability before the innovation happened’ up to 5.67 for ‘Individual leadership’.

**Table 8:** Summary of significant correlations between factors enabling or hindering the most economically important governance innovation types.

* Correlation is significant at the 0.05 level (2-tailed); ** Correlation is significant at the 0.01 level (2-tailed)

Correlation based on the results from Pearson’s correlation matrix: measured as a point-biserial correlation matrix between the factors enabling or hindering the most economically important innovations and governance innovation types. Blue color symbolizes a positive correlation between the factors and innovation types (enabling), red color indicates a negative correlation (hindering factors). Only significant correlations between variables are presented in this table.

‘Climate change’ and ‘Knowledge available’ arise as strong enabling factors that contribute to ‘New trans-sectoral contracts created’ (Table 8). ‘Climate change’ together with ‘Culture of your organisation’ were seen as factors attracting ‘New users of ecosystem service(s)’. Further, ‘High profitability/viability before the innovation happened‘ and ‘Private sector and business’ are particularly enabling the development of ‘New technology for biomass production’ whereas ‘Low profitability/viability before the innovation happened‘ is hindering these innovations. Negative correlations were found between ‘Change of forest management to improve/sustain biomass production’ and ‘Individual leadership’ as well as between ‘High profitability/viability before the innovation happened’ and ‘Change of forest management to provide other ecosystem services’.

### 4.4 Influence of forest ownership and size on governance innovation development

An exploratory analysis of the influence of forest size and ownership types on governance innovation revealed that ‘Public ownership by state at national level’ is the least represented ownership type, but represents the larger forest properties (> 470 ha). ‘Private ownership by individual or family’ is the most represented ownership type in the survey, but represents the smallest forest properties (0–8 ha) (Supplementary Material Fig. S4).

**Fig. 1:**
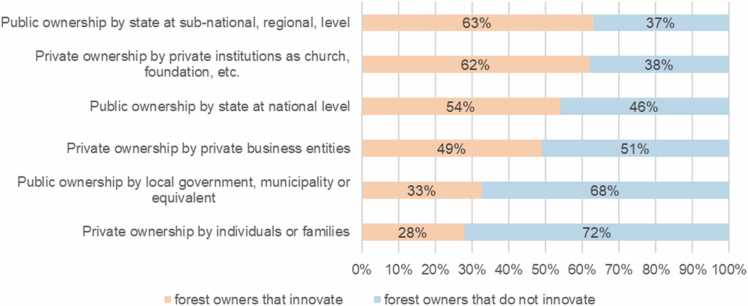
Development of innovations by forest ownership types.

Relating innovation development to ownership types, we found that ‘Public ownership by state at sub-national, regional level’ and ‘Private ownership by private institution as church, foundation, etc.’ develop governance innovations more often (> 60%) than ‘Public ownership by local government, municipality or equivalent’ and ‘Private ownership by individuals or families’ (< 35% each) (Fig. 1).

**Fig. 2:**
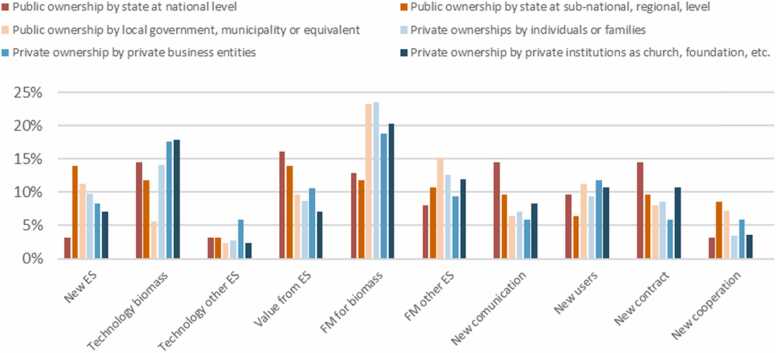
Types of innovation (in percentage) implemented by the different ownership types.

Analysing patterns of the relationships between forest ownership types and governance innovations, it appears that all forest ownership types developed ‘New technologies for biomass production’. Inversely, ‘New technologies for other ecosystem services’ are commonly less developed by forest owners and managers. The focus on biomass production was also reflected in innovations that target forest management practices, i.e. ‘Change of forest management to improve/sustain biomass’ is commonly more applied compared to ‘Change of forest management to provide other ecosystem services’. Observing the general shapes of the curves, public national and regional forest owners have rather comparable innovation strategies that differ from innovation strategies of other ownership types. Moreover, innovation strategies of public forest owners at the local/municipality level seem to be closer to those of private forest owners, with few exceptions (Fig. 2).

**Fig. 3:**
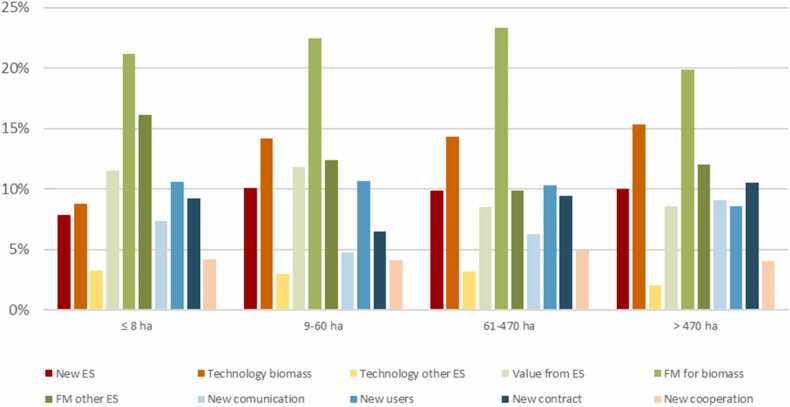
Types of innovation (in percentage) implemented in different sizes of forest properties.

Forest size also correlated with governance innovation development. In general, forest owners appeared to engage in innovation activities to improve/sustain biomass production rather than to provide other ecosystem services, independently of the size of the forest (Fig. 3). Owners of small forest properties showed comparatively lower engagement for new technologies that support biomass production compared to owners with larger properties. However, we also found forest owners with smaller properties who innovated more in terms of changing forest management to provide other ecosystem services than owners with larger forest properties.

## 5 Discussion

### 5.1 Discussion of findings

Our analysis of forest owners and managers developing governance innovations for FES provision offers insights on the social, economic, and ecological challenges the European forestry sector is currently facing. A central finding is that innovation activities are largely linked to biomass production. Most forest owners and managers - private like public – implement efficiency-driven optimisation strategies to improve or sustain biomass production to generate income. This underlines that forestry related innovations largely continue the long history of focusing on material aspects of forestry through developing effective silvicultural practices (Puettmann et al., 2012) to satisfy respective local (Elbakidze and Angelstam, 2007) or industrial needs, and create an effective wood-based value chain (Melnykovych et al., 2018, Prokofieva and Wunder, 2014).

The focus on biomass related innovations is understandable given the underlying economic rationale of provisioning services (Lindahl et al., 2017) and the public or common good character of many regulating and provisioning FES resulting in positive external effects. The latter makes it difficult to trade them at markets (Muradian and Rival, 2012) and to incorporate them in ‘innovation strategies‘ of forestry companies oriented towards profits or financial stability. As increasing provisioning services can reduce the provision of regulating and cultural FES (Hauck et al., 2013), conflicts emerge over forest uses in particular between production and conservation functions and services (e.g., Thorsen et al., 2014; Kleinschmit et al., 2017; Sotirov and Winkel, 2016), and cultural FES such as recreation (Bauhus et al., 2017b, Tyrväinen et al., 2017). Even though regulating and cultural FES are promoted in various national and international policy agendas such as the EU Green Deal, the Biodiversity Strategy, and the EU Forest Strategy (Wolfslehner et al., 2020), the development of policy instruments at local-regional level, strategic and tactical planning, and operational management that promote ecological, social, and cultural forestry objectives lag behind (Angelstam et al., 2018, Lindahl et al., 2017). Despite a substantial interest of forest owners and managers in regulatory (Maier and Winkel, 2017, Winkel et al., 2015) or cultural FES (Torralba et al., 2020b) and recognition of their importance, the challenge to align the innovation perspectives of forest owners and managers with such policy demands remains.

Our findings hence point to the necessity to support forest owners and managers in achieving a more diversified portfolio of forests and forest operations toward broader bundles of ecosystem services supply. Such diversification is also in the interest of an increased resilience of forests to future social-ecological shocks, such as those imposed by climate change. More specifically, in our view, pathways towards a broader spectrum of ecosystem services needs coherent action both from forest operations and from public policies. Regarding forest owners and managers, using a system-based approach to understand the forestry contexts for innovation allowed us to gain insights into required context conditions for action. In particular, private forest owners and businesses whose innovation practices increase provisioning services with targeted management and market strategies and infrastructures are open to innovations. In addition, climate change and related adaptation needs are seen as an enabling factor for – or enforcing – innovations referring to forests carbon sequestration and mitigation potentials (Bowditch et al., 2020, Jordan and Huitema, 2014). However, the low profitability of other FES largely hinders innovation development in the forestry sector. For their provision, changes are needed on individual and institutional levels with help of governmental and state interventions.

On an individual level, individual leadership seems a crucial factor for ‘out of the box’ innovations (i.e. innovations with other FES), while changes in forest management practices for improved biomass provision are negatively correlated with individual leadership. One might interpret this in a way that the path dependencies of the ‘classical’ forestry regime with its focus on optimizing biomass production are too strong and preventive to changes (Lindahl et al., 2017). Thus, requiring even more leadership and respective knowledge to explore new business or activities relating to new FES, niche innovation development, testing, and momentum for successful change (Geels, 2011). Forest owners’ responses indicate that in particular cultural FES are addressed with new communication and marketing strategies, and the identification of new users is a precondition for such service provision. These kinds of innovations require changes not only on an individual level but also in “the culture of organisation”, to be open towards societal demands. Coordinated action and mechanisms to “open-up” and “broaden out” problem perceptions and solution development as well as to make necessary tradeoffs explicit seems key (Karpouzoglou et al., 2016, Lindahl et al., 2017, Meier et al., 2016).

Forest owners and managers could introduce new business models that capitalize on those ecosystem services for which demand outmatches supply. In particular, there is a large societal interest in foraging wild natural resources, such as mushrooms and berries, in sports- and health-related outdoor recreation, and in environmental and forest-related education, and substantial amounts of investment are made in these areas. For instance, spending of 23 million anglers, 7 million hunters and 6 million birdwatchers had been estimated to amount to 40 billion € in 2006 (€121 per ha of land in the EU) (Kenward et al., 2009). Forest enterprises have rarely participated in this creation of value based on biodiversity and ecosystem services so far.

On an institutional level, changes in demand structure for FES need to be accompanied by benefit transfers to FES providers before investments into innovation activities are considered. For governance innovations, two pathways for action are supported by our analysis findings: one option is the design of new trans-sectoral contracts between public and private forest owners for better aligning FES demand and supply. These might directly link up to climate change pressures as a ‘hook’ to support forest owners in changing their management focus as well as to respond to growing socio-political demands for regulating services that require the integration of new knowledge to overcome knowledge gaps. Examples are public-private partnerships for linking forest management with tourism demands and recreation activities or with nature conservation initiatives (Abruscato et al., 2020, Thellbro et al., 2018). The other option is to advance with payments for ecosystem services (PES). Research in other contexts showed that PES and other incentive-based instruments could foster the provision of regulating and cultural FES. However, their design and implementation are challenging. Issues such as trust, fairness, and others’ perceptions may play a crucial role in the process of establishing payment schemes (Loft et al., 2017, Primmer et al., 2014, Prokofieva and Wunder, 2014). Many PES programs reinforced conflicts over access and control over forest resources (Corbera et al., 2007; Sconfienza, 2017). To encounter this, a growing body of literature related to PES (e.g., Alpízar et al., 2017; Ferraro and Hanauer, 2014) and other incentive programs (e.g., Ashraf et al., 2014) points out that building non-monetary decision-making preferences into policy instruments can increase conservation efforts and people’s satisfaction with the transaction. Conversely, failing to do so can have unintended negative effects (Loft et al., 2020; Loft et al., 2015).

While various innovative governance models have been developed that connect forest owners and managers to this societal demand (Mann et al., 2021), innovation development may be associated with establishment and development costs. These costs do for example occur for a change of management to provide other FES or the identification of new user groups, and may prevent innovation. Recognizing that about half of the responding forest owners and managers have indicated that they do innovate to provide other FES, to generate value or to identify new users, more advanced forms of policy instruments, operational management, and financing schemes rooted in close communication and cooperation among stakeholders seem to be needed in order to foster this trend. Building on intersectoral policy frameworks such as the European Green deal or the One Health approach may allow for funding streams from policy sectors such as health, sports, youth, culture, integration or climate on national to local level.

Finally, our results indicate the influence of forest ownership types and size on innovation activities. Land tenure appears to be more relevant for innovations than forest size. There is a tendency that private forest owners focus on innovations related to biomass production while the public sector seems more active in innovations for FES diversification. Given the high share of private forests in Europe, this finding is important for formulating policy recommendations (Nichiforel et al., 2018). Knowing that a lack of formal rules for financing, collaboration and contracts are perceived as burdens for FES provision in practice, these conditions require improvement for the private forestry sector to stronger convert towards multiple FES provision. In contrast, public forest owners show a higher attitude towards innovation development for new ecosystem services. This is not surprising due to the common welfare orientation of public forests in general (Ruppert-Winkel and Winkel, 2011; Sotirov et al., 2014), and chance for experimenting and diversifying forest products and services on large scale.

### 5.2 Discussion of methods

The collected sample does not statistically represent the population of forest owners and managers in Europe, in particular not in terms of geographical origin and coverage of forest area. In addition, the sample displays a non-normal distribution of forest ownership and sizes. The main reason for this is the lack of a comprehensive European database of the total population of forest owners and managers, next to limited possibilities to access them. The chosen non-random pyramid and snowball sampling via umbrella-type organizations was inevitable to encompass this social system (Atkinson and Flint, 2004) and to reach as many types of forest owners and managers across countries as possible. For the same reason the actual return rate cannot be calculated. The main practical implications are on the one hand that the final sample is dominated by countries, organisations, and individual respondents that are more active in the field than the assumed average (ibd.). On the other hand, these distribution channels limited contacts to ‘non-traditional’ (also termed urban or new) forest owners as these are less institutionally organized (e.g., Joa and Schraml, 2020 for Germany; Hirsch and Schmithüsen, 2010 for Europe). This may cause a certain bias on our data towards larger and more active, i.e. also economically oriented forest owners and managers, thus possibly the potential for exploiting synergies in diversifying the portfolio for FES provision tapping into forest owners not primarily interested in the provision of biomass may be underestimated in this paper (cf., Winkel, 2006). We argue, however, that the chosen distribution mode and the sample obtained is still valuable for a quantitative exploratory study on innovation activities and decisive conditions in forestry. The overall return from all 17 countries included representation of all forest ownership types, forest sizes, governance innovations and FES provided. This enabled a statistical analysis within these variables in spite of the sample’s geographical focus on three central and northern European countries (cf. Grossmann et al., 2020).

Because of the lack of a sufficiently large sample size, we could not develop a regression model (binary logistic regression). The non-normal distribution of data restricted our analysis to exploratory statistics to see general trends in data distribution, factor analysis results and correlation matrices. Other methods applied that might have allowed developing a binary logistic regression model to gain more information about relationships between variables and their influence did not prove successful. All this limits the representativeness of our findings. However, our findings do provide a snapshot of forest owner perceptions and attitudes towards governance innovations for FES provision. Their willingness to change forest management regimes for more sustainable and widespread FES provision becomes a crucial adjustment screw in times of dealing with grand societal challenges. Prospectively forests become more and more a central part of the solution to encounter climate change and biodiversity loss, but its potential is not yet sufficiently recognised, communicated, and valorized.

Further information on national geographic and socio-political influences on multifunctional forest management in different European countries is provided e.g. by Elands and Wiersum (2003), and insights into the distribution of private forest owner structures and their demographic profiles (gender, age, full and part time employment of private forest owners) in different European countries by Hirsch and Schmithüsen (2010). An alternative to obtain a more comprehensive European overview of FES-related governance innovations is be to perform a qualitative comparative study based on a large number of interviews with forest owners and managers which was beyond the scope of our study. In this regard, the study of Matilainen et al. (2019) can serve as a promising starting point for further qualitative elaborations.

## 6 Conclusions

Innovations in the European forestry sector to sustain FES are scarce and scattered, in particular for regulating and cultural services. The main obstacle for the latter is the reliance of forestry on a market-oriented economic rationale for biomass production that reinforces a timber production-oriented forest management paradigm. Due to the lack of competitive options for generating income, innovators are directed towards biomass production where the market exists. The lack of options to generate sustained income with other FES that would provide backup and security to forest owners and managers to engage in related governance innovation development reinforces the orientation of forest managers and also related forest policy makers to defend the timber primacy system (Sotirov and Winkel, 2016). This poses a dilemma and makes it more difficult to diversify FES related forest management activities and innovations.

We see in our analysis, however, that forest owners and managers perceive societal demand for improved ecosystem service delivery, but their supply requires institutional support that allows for needed transformations. On EU policy level, currently the Green Deal as well as revisions of the Forestry Strategy offer windows of opportunity to better foster FES provision on a European scale. More than before are forests at the heart of solution strategies for biodiversity conservation and climate change adaptation. These political quests need to become materialized for private and public forest owners to acknowledge and compensate for their additional efforts for FES provision. What becomes visible is that currently mainly public forests undertake innovation activities for better service provisioning, while large parts of the private forest owners innovate largely only in relation to biomass production, following established market incentives. Considering the large share of forest area in Europe in private hands, leaving these actors out of the solution process is a lost opportunity. Prospectively the provision of biodiversity habitat, carbon sequestration, and recreation services should be an explicit part of the forestry portfolio and a management alternative where the EU provides a framework with a forestry strategy that helps to align actors and sectors for sustainable forests. It is promising that we find many good examples of innovations and active forest owners and managers all over Europe that successfully provide a range of FES according to socio-political demands. These can serve as good practice examples for exchange and learning among scientists, practitioners and policy makers to showcase functioning innovation development and to increase innovation activities in forestry across Europe.

## Declaration of Competing Interest

The authors declare that they have no known competing financial interests or personal relationships that could have appeared to influence the work reported in this paper.

## Footnotes

1 https://maptionnaire.com/
