# Supplementary material for: Governance Innovations for forest ecosystem service provision – Insights from an EU-wide survey
Source: Environ Sci Policy. 2022 Jun;132:282–95. doi: 10.1016/j.envsci.2022.02.032 (PMC8996823; doi:10.1016/j.envsci.2022.02.032)
Supplement: Supplementary file 1 — Supplementary material [file mmc1.docx]

# Annex

**A) Full survey**

[**https://app.maptionnaire.com/en/5199/**](https://app.maptionnaire.com/en/5199/)

**B) Classification of Forest ecosystem services addressed in the survey**

| Category | Sub-Category | Examples |
| --- | --- | --- |
| Provisioning | Biomass (wood) | Fibres, wood, timber for material use |
|  |  | Wood for energy use |
|  | Game | Hunting |
|  | Wild forest products | Berries, mushrooms, nuts, medicinal plants |
| Regulating | Watershed protection | Water and erosion control |
|  | Air quality regulation | Filtration, pollutant sequestration |
|  | Climate change mitigation | Carbon sequestration and storage |
|  | Habitat for plants and animals | Habitat provision and biodiversity, incl. pollinators or seed dispersal forest species |
| Cultural | Cultural, emotional and spiritual values |  |
|  | Education | Forest kindergarten, schools |
|  | Healthcare, sports and outdoor recreation | Nature-based tourism |

**C) Conversion of continuous scale (1-100) to a 7-point Likert scale**

| Likert scale code | Range | Conceptual interpretation |
| --- | --- | --- |
| 1 | 1-14 | very strongly not supplied/demanded |
| 2 | 15-29 | strongly not supplied/demanded |
| 3 | 30-43 | not supplied/demanded |
| 4 | 44-57 | neutral (discarded in analyses) |
| 5 | 58-71 | Supplied/demanded |
| 6 | 72-86 | strongly supplied/demanded |
| 7 | 87-100 | very strongly supplied/demanded |

**Supplementary material**

All Tables S1-S9 and Figures S1-S4 can be found in the Supplementary material under the link:

<https://nextcloud.hnee.de/s/9tBCaMRZyPFARkH>
